# Establishing Reference Genes for Accurate Gene Expression Profiling in Toxigenic *Bacillus cereus*

**DOI:** 10.3390/toxins17020058

**Published:** 2025-01-27

**Authors:** Tanja V. Edelbacher, Astrid Laimer-Digruber, Michael W. Pfaffl, Monika Ehling-Schulz

**Affiliations:** 1 Institute for Microbiology, University of Veterinary Medicine Vienna, 1210 Vienna, Austria; t.edelbacher@umcutrecht.nl (T.V.E.); astrid.laimer-digruber@vetmeduni.ac.at (A.L.-D.); 2 Division of Animal Physiology and Immunology, School of Life Sciences Weihenstephan, Technical University of Munich (TUM), 85354 Freising, Germany; michael.pfaffl@tum.de

**Keywords:** *Bacillus cereus*, human pathogen, reference genes, toxin gene expression, RT-qPCR, targeted gene expression analysis

## Abstract

*Bacillus cereus* is a Gram-positive pathogen associated with foodborne illnesses and severe non-gastrointestinal infections. Robust tools for accurate gene transcription analysis are essential for studying toxin gene expression dynamics and deciphering the complex regulatory networks orchestrating the expression of toxin and virulence factors. This study aimed to identify reliable reference genes for normalizing reverse transcription quantitative PCR (RT-qPCR) data in toxigenic *B. cereus*. An emetic and an enteropathogenic strain were used as model organisms to establish a suitable reference gene set to monitor the dynamics of toxin gene transcription. Ten candidate reference genes were evaluated for their expression stability using geNorm, NormFinder, BestKeeper and the ΔCq method, with the final rankings integrated via RefFinder. Among the tested genes, *rho*, *rpoD* and *recA* were identified as the most stable expressed reference genes across all tested conditions. As shown in this proof-of-principle study, the established reference gene set provides a suitable tool to investigate the influence of extrinsic and intrinsic factors on toxin gene transcription. In conclusion, our newly established reference gene set provides a robust basis for studying toxin gene expression in *B. cereus* and contributes to a better understanding of its pathogenicity and potential strategies to mitigate its harmful effects.

## 1. Introduction

*Bacillus cereus* is an endospore-forming Gram-positive pathogen known for its association with foodborne illnesses and gastrointestinal infections [1,2,3,4,5]. Recent evidence has linked *B. cereus* to a broader spectrum of acute non-gastrointestinal diseases, such as sepsis and central nervous system infections, particularly in immunocompromised patients and neonates [6,7,8,9,10,11]. The pathogenicity of emetic *B. cereus* can be attributed to its emetic toxin, cereulide—a cyclic dodecadepsipeptide known for its resistance to heat and gastric acid, which can cause severe symptoms ranging from vomiting to organ failure [12,13,14,15]. In addition, the diarrheal syndrome is mediated by multi-component pore-forming enterotoxins, such as the non-hemolytic enterotoxin (Nhe) and hemolysin BL (Hbl) [16,17,18].

Cereulide acts as a potassium ionophore. Its enzymatic synthesis is directed by a non-ribosomal peptide synthetase (NRPS) system encoded by the *ces* gene cluster (*cesHPABCD*) [2,3,19,20]. While the influence of growth conditions on cereulide production has been studied [21,22,23], the transcriptional regulation of *ces* gene expression warrants further investigation to elucidate the complex regulatory network governing cereulide biosynthesis fully.

The Nhe enterotoxin complex is ubiquitous among enteropathogenic *B. cereus* strains.

Studies have shown that it induces pore formation and subsequent cell membrane disruption in vitro [24], governed by the interaction of three components: NheA, NheB and NheC [25,26,27]. The B. cereus phospholipase sphingomyelinase (SMase), encoded by sph, is another important factor that interacts synergistically with Nhe and Hbl toxins to exacerbate disease severity [28,29]. By hydrolyzing sphingomyelin, B. cereus SMase influences membrane dynamics and modulates the host immune response [16,30,31]. In addition to SMase, there are other phospholipases, such as the phosphatidylcholine-specific phospholipase C (PC-PLC), encoded by the gene plc, that target lipids of eukaryotic and prokaryotic membranes, which might drive B. cereus-induced endophthalmitis [32,33,34]. Among other virulence factors, Nhe, SMase and PC-PLC are regulated by the pleiotropic transcriptional regulator PlcR [35,36,37,38,39].

Various growth media have been developed to cultivate bacteria, depending on the species and the study’s objectives. It has been shown that nutrient availability influences toxin production in several microorganisms, with nutrient limitation affecting the production of certain virulence factors [40,41,42,43]. Chemically defined minimal media have been developed to determine the essential nutrients that are critical not only for sustaining growth but also for meeting the nutritional requirements of enterotoxin synthesis [44,45]. In this study, we compare the commonly used LB-Miller broth according to Miller et al. (LB) [46], which is a rich medium, with the modified optimal defined medium MOD [45], which is frequently used to mimic host conditions. To understand the complex mechanisms behind disease etiology, it is necessary to develop a robust framework for fundamental in vitro studies. Currently, research is hampered by the lack of established reference genes for normalizing reverse transcription quantitative PCR (RT-qPCR) data in toxigenic *B. cereus* that can be shared and commonly used by researchers in the field. Therefore, we aimed to advance and standardize RT-qPCR methods by identifying and validating stable expressed reference genes for toxigenic *B. cereus*.

State-of-the-art RT-qPCR data analysis requires the use of reference genes with stable expression across different conditions to reduce sample variability and improve reproducibility [47,48,49]. For instance, reference genes must be stably expressed when cultivated in different media at different growth stages. Due to the versatility of metabolic phenotypes, cultivation conditions of bacterial organisms and the diversity of bacterial species, reference genes need to be evaluated for suitability in different experimental settings [48,50,51]. Furthermore, RT-qPCR data acquisition relies on technical accuracy, which is affected by different input amounts of genetic material, variances in RNA integrity and reproducibility between different RT-qPCR setups. This additionally necessitates the identification of stably expressed reference genes, to elucidate intricate biological processes that would otherwise be overshadowed by technical variabilities [50,52].

Due to the strong demands from the scientific community and the rise of transcriptional studies, several models have been proposed and developed to determine the most stable reference genes. The most prominent tools include BestKeeper [47], NormFinder [53], geNorm [54] and the comparative ΔCq method [55]. As they are based on different mathematical algorithms, stability rankings can differ between the models. Therefore RefFinder was developed to provide a holistic overview of the different stability rankings and to facilitate comprehensive, data-driven decision making [56].

We hypothesize that stable reference genes can be established for different types of toxigenic *B. cereus* that allow monitoring of toxin gene and virulence factor expression in relation to different growth phases and cultivation media. Thus, an RT-qPCR assay was employed to identify suitable reference genes, which were subsequently applied as a proof of principle to study toxin gene expression of toxigenic *B. cereus* strains across different growth phases and media conditions.

## 2. Results

### 2.1. Candidate Reference Genes for RT-qPCR Studies in Eubacteria

The diverse nature of bacterial species and the different means of cultivation exacerbate the challenges of genetic circuit analyses. RT-qPCRs are a widely used and resourceful tool to address various biological questions beyond the purpose of untargeted transcriptomic analysis. Despite many studies being aimed at establishing effective reference genes for different biological systems, the diversity among bacterial species still poses a challenge in RT-qPCR-based studies.

**Figure 1:**
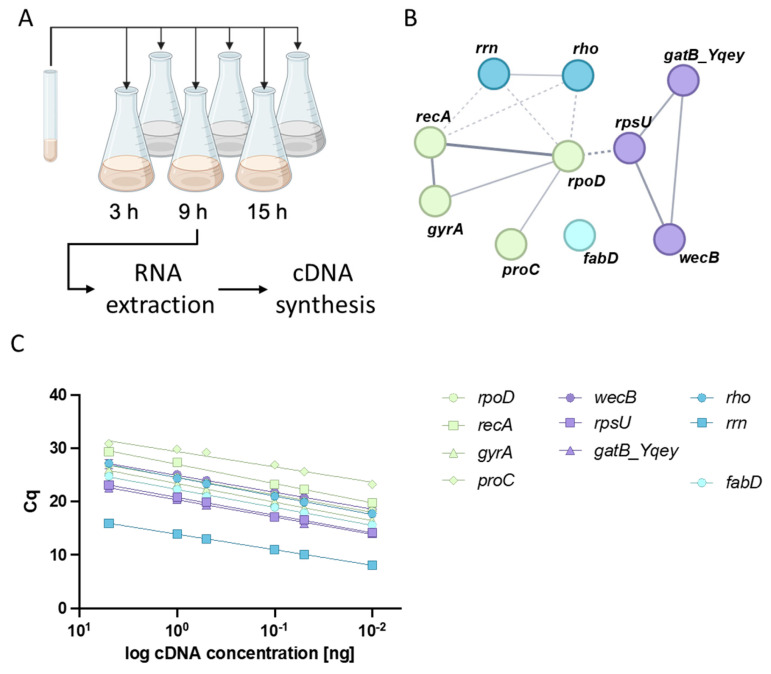
Schematic overview of identification of suitable reference genes and primers for RT-qPCR analysis of toxigenic *B. cereus*. (**A**) Cultivation of bacteria: 100 mL main cultures, either LB or MOD, inoculated with *B. cereus* precultivated in 3 mL LB overnight at 30 °C. Start OD_600_: 0.05. For details, see Material and Methods (created with biorender.com); (**B**) bioinformatical network analysis (STRING DB) to identify putative protein–protein interactions of the gene products of candidate reference genes; (**C**) RT-qPCR efficiency: slopes of primer sets targeting candidate reference genes were used to calculate RT-qPCR efficiencies.

We reviewed the literature on bacterial reference genes for the *B. cereus* group [57], *Klebsiella pneumoniae* [58] and *Staphylococcus aureus* [59] to identify potentially suitable reference genes, which allow us to investigate the dynamics of toxin gene and virulence factor expression in toxigenic *B. cereus* in different environments (Figure 1A). This approach requires reference genes that are not affected by nutrient availability or growth phase. Candidate reference genes identified by the literature review were classified according to their biological function using the STRING database (https://string-db.org, accessed on 20 November 2024, Figure 1B) [60]. This approach revealed the following genes as possible reference candidates, which were tested for suitability and robustness: *rpoD* (RNA polymerase sigma factor RpoD), *fabD* (Malonyl CoA-acyl carrier protein transacylase), *rho* (Transcription termination factor), Rho*proC*, (Pyrroline-5-carboxylate reductase), *gyrA* (DNA Gyrase subunit A), *wecB* (UDP-N-acetylglucosamine 2-epimerase), *gatB_Yqey* (gatB/Yqey domain-containing protein), *rpsU* (30S ribosomal protein S21), *rrn* (16S rRNA) and *recA* (Recombinase A) (for details of reference candidate genes, see Table S1).

Although all genes, except *fabD*, are directly associated with at least one other gene, they can still be grouped into different subclasses (Figure 1B). For each of these genes, primers were designed specifically for pathogenic *B. cereus* and primer efficiencies were calculated (Figure 1C, Table 1). Two strains originating from foodborne outbreaks, the emetic reference strain F4810/72 and the enteropathogenic reference strain NVH0075-95, were used to test the suitability of the candidate reference genes for normalizing RT-qPCR data. Except for *proC*, no significant differences in the Cq values for the tested candidate reference genes were observed between the two strains grown in two different media (see Figure S1).

**Figure 2:**
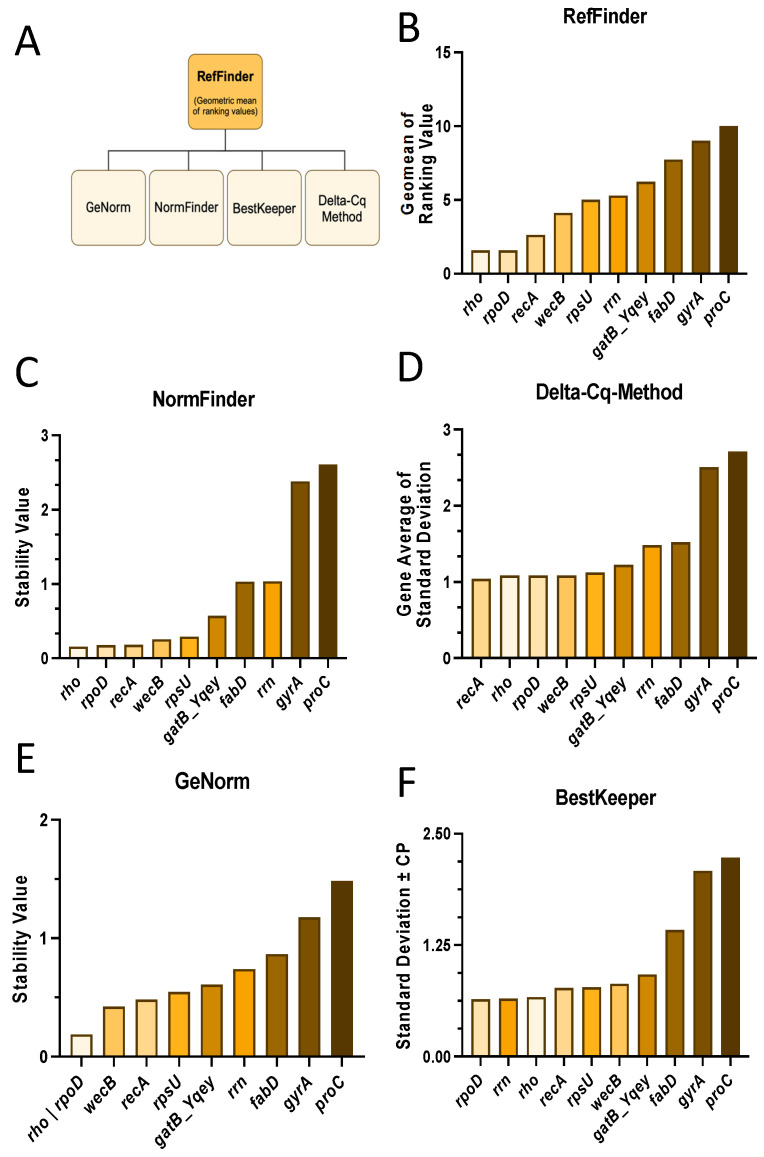
Validation of reference genes for transcriptional studies of toxigenic *B. cereus*. (**A**) The scheme shows four common mathematical models to identify reference genes (created with biorender.com), (**B**) which are summarized by their geometric mean by RefFinder. Rankings of each model are shown for (**C**) NormFinder; (**D**) comparative ΔCq; (**E**) geNorm; and (**F**) BestKeeper.

Currently, several methods have been proposed to identify reference genes, and RefFinder can be utilized to obtain a comprehensive ranking of different algorithms, integrating results from geNorm, NormFinder, BestKeeper, and the comparative ΔCq method (Figure 2A) [56]. The comprehensive ranking of the candidate reference genes indicates that *rho*, *rpoD* and *recA* are the three most stable genes (Figure 2B). While the ranking of the least stable genes (*gyrA*, *proC*) is consistent across all four models, the most stable genes differ (Figure 2C–F). For NormFinder (Figure 2C) and the comparative ΔCq method (Figure 2D), the top three most stable genes—*rho*, *rpoD*, *recA*—correspond to the top three genes in the comprehensive ranking (Figure 2B). In contrast, GeNorm ranks *wecB* third and *recA* fourth (Figure 2E), while BestKeeper places *rrn* in the second position (Figure 2F), although all the other three models rank it in the lower half (Figure 2C–E).

**Figure 3:**
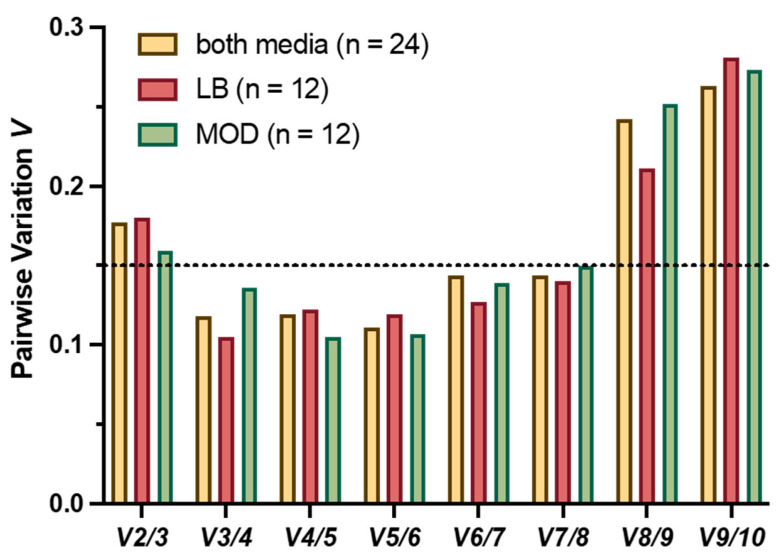
Pairwise variation (V) of *B. cereus* candidate genes. The pairwise variation (V_n_/V_n+1_) was calculated between the consecutive normalization factors NF_n_, and NF_n+1_ using geNorm to determine the optimal number of reference genes. The dotted line indicates the threshold of 0.15, below which including additional reference genes is not necessary.

The GeNorm algorithm was also used to determine the minimum number of reference genes required for adequate normalization of the target genes by a pairwise variation (V) analysis. Three genes were found to be sufficient for adequate normalization across the entire data set with two cultivation media and three time points each, as the pairwise variation value is below the commonly accepted threshold of 0.15 (Figure 3) [18]. For data normalization in subsequent assays, the estimated number of three genes required for data normalization was combined with the comprehensive ranking from RefFinder, resulting in the reference gene combination *rho*, *rpoD* and *recA*.

### 2.2. Distinct Toxin Gene Expression Pattern in Emetic and Enteropathogenic B. cereus

**Figure 4:**
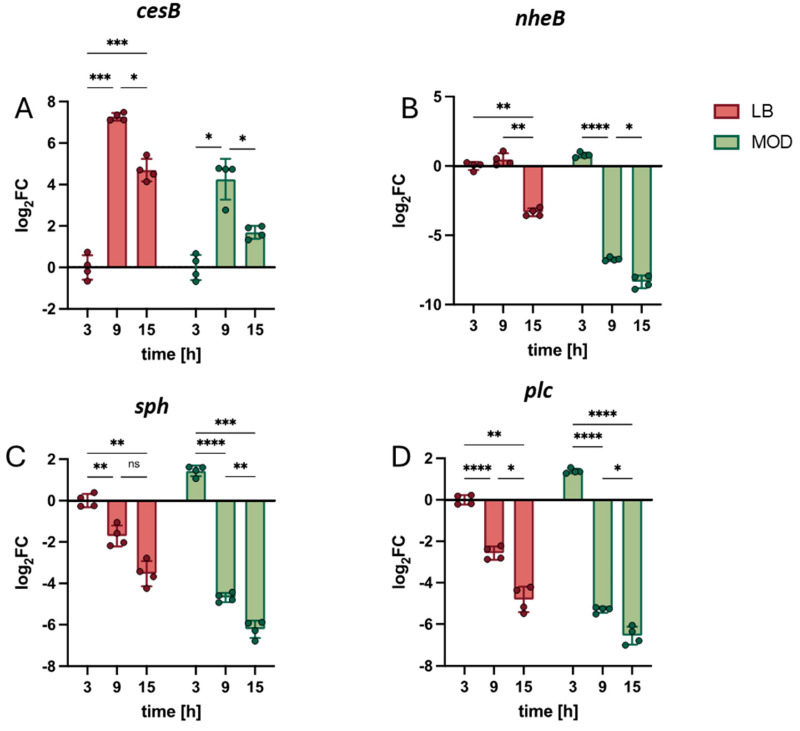
Spatiotemporal analysis of toxin gene expression in emetic *B. cereus* using the newly defined set of reference genes. Dynamics of toxin gene transcription of (**A**) *cesB*, (**B**) *nheB*, (**C**) *sph* and (**D**) *plc* are shown for emetic *B. cereus* (F4810/72) grown in LB and MOD. Samples were taken after 3 h, 9 h and 15 h incubation at 30 °C, 120 rpm. The most appropriate combination of constitutively expressed reference genes—*rho*, *rpoD* and *recA* (see Figure 2)—was used to normalize the data. To assess transcriptional patterns, each condition was further normalized to gene expression of bacteria grown in LB for 3 h. Statistical significance was calculated by two-way ANOVA with Tukey‘s correction for multiple testing. Statistical significance levels are indicated as follows: ns > 0.05, * *p* < 0.05, ** *p* < 0.01, *** *p* < 0.001, **** *p* < 0.0001.

The combined action of different toxins characterizes pathogenic *B. cereus*. Therefore, the expression of the key toxin encoding genes *cesB*, *nheB*, *sph* and *plc* was assessed. In general, all conditions were normalized to the three-hour timepoint in LB. For *cesB*, which showed a similar gene expression pattern in both LB and MOD, gene expression peaked at nine hours (Figure 4A). For *nheB*, the gene expression remained constant between three and nine hours in LB but decreased significantly at 15 h. In MOD, *nheB* expression started to decrease already after nine hours (Figure 4B). The expression of the phospholipase genes *sph* and *plc* follow similar patterns, characterized by a continuous decrease in gene expression levels over time in both media (Figure 4C,D).

However, the differences are more pronounced in MOD with a significantly greater decrease in expression levels over time (e.g., *sph* t_9_-t_3_ LB −1.71 ± 0.50, MOD: −6.13 ± 0.24; *plc* t_9_-t_3_ LB −2.57 ± 0.27; MOD: −6.69 ± 0.13). Furthermore, *sph* and *plc* gene expression in MOD is higher compared to LB at three hours (Figure 4C,D).

**Figure 5:**
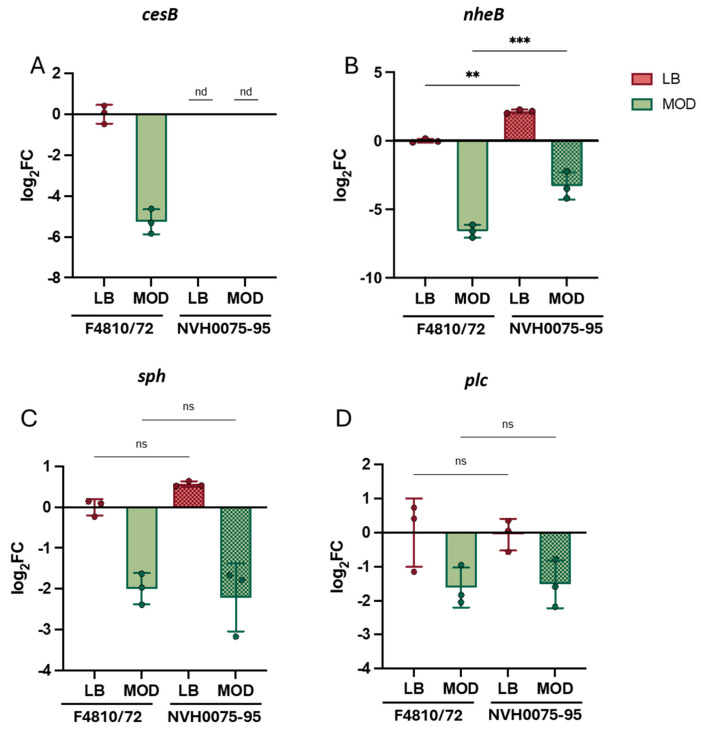
Comparative transcriptional analysis of toxin genes in emetic and enteropathogenic *B. cereus*. Toxin gene expression of (**A**) *cesB*, (**B**) *nheB*, (**C**) *sph* and (**D**) *plc* are shown for emetic (F4810/72) and enteropathogenic (NVH0075-95) *B. cereus* grown in LB and MOD at 30 °C for 9 h. The newly established set of reference genes, *rho, rpoD* and *recA* (see Figure 2), was used for data normalization. Each tested condition was further normalized to the gene expression of the emetic strain (F4810/72) grown in LB. Statistical significance was calculated by one-way ANOVA with Tukey‘s correction for multiple testing. Statistical significance levels are indicated as follows: ns > 0.05, ** *p* < 0.01, *** *p* < 0.001; ‘nd’ indicates not detected.

To determine whether toxin gene expression differs between emetic and enteropathogenic *B. cereus* strains, RT-qPCR was performed after nine hours of cultivation. A separate set of samples containing both bacterial strains was used to account for batch-to-batch variation. All gene expression data were normalized to the corresponding gene expression of F4810/72 in LB. As cereulide production is limited to the emetic *B. cereus*, no *cesB* transcripts could be detected in the enteropathogenic NVH0075-95 (Figure 5A). For *nheB*, strain-specific differences were observed in both LB and MOD, with *nheB* expression levels being significantly higher in the enteropathogenic NVH0075-95 (Figure 5B). The phospholipases *sph* and *plc* did not show any strain-specific differences, despite media-dependent regulation of gene expression (Figure 5C,D).

## 3. Discussion

*B. cereus* is a Gram-positive, soil-dwelling endospore-forming human pathogen implicated in the causation of foodborne outbreaks and acute infections [2,3]. Its pathogenicity is linked to several virulence-related genes that determine the disease development [16,61]. The intricate mechanisms regulating toxin production have not yet been fully elucidated. Thus, the aim of the present study was to identify constitutively expressed reference genes suitable for normalization of RT-qPCR gene expression data in different growth phases and cultivation conditions of toxigenic *B. cereus* strains. Reliable normalization of RT-qPCR data is based on the use of stable reference genes, which demands testing candidate reference genes under different experimental conditions [48,50,51]. Although 16S-RNA has been widely used for data normalization and has therefore been extensively studied and discussed, it is generally acknowledged that *rrn* is not suitable for RT-qPCR data normalization [58,59,62,63,64]. Indeed, corroborating previous findings, our survey showed that the 16S rRNA gene (*rrn*) is not found to be among the most stable expressed genes in *B. cereus* under our tested conditions. Thus, other reference genes are needed to gain in-depth insights into the dynamics of toxin gene and virulence factor transcription in bacterial pathogens under varying environmental conditions.

### 3.1. Identification of Candidate Reference Genes for RT-qPCR Studies in Eubacteria

Based on a thorough literature review, we compiled a list of ten genes, which we tested for their potential to serve as references for transcription analyses of key virulence factors and toxins in B. cereus. Our study revealed that the previously proposed reference genes *gatB_Yqey*, *rpsU* and *wecB* [47], are not the most suitable ones for transcriptional studies in toxigenic *B. cereus* as the reference genes *rho*, *rpoD* and *recA* outperform them. Although the cultivation conditions in our study were very distinct and drove bacterial metabolisms into different states, the identified genes *rho*, *rpoD* and *recA* were proven suitable for data normalization. Notably, the genes we identified as the most suitable reference genes for studying pathogenic *B. cereus* are also the ones previously reported to be the most stable expressed genes in other bacterial species, such as *Acinetobacter baumannii* [62], *Staphylococcus pseudintermedius* [65], *Klebsiella pneumonia* [58], *Staphylococcus aureus* [59] and *Clostridium perfringens* [63]. Since they all have an important role in the RNA machinery [66,67,68], it is tempting to speculate that these genes are constitutively expressed across the bacterial kingdom and could serve as commonly used (or even universally used) reference genes in Eubacteria.

### 3.2. Distinct Toxin Gene Expression Pattern in Emetic and Enteropathogenic B. cereus

The emetic type of *B. cereus* is characterized by the presence of the *ces* gene cluster, which is located on the pXO1-like plasmid pCER270 [19,20]. The *ces* genes encode the CesNRPS, a mega multienzyme machinery responsible for the assembly and export of the depsipeptide toxin cereulide [19,69]. While the *ces* genes are only found in emetic *B. cereus*, the genetic determinants involved in the enteropathogenicity of *B. cereus* are commonly found among all *B. cereus* group members [6,70]. Our newly established set of reference genes was successfully employed to study toxin gene expression patterns in an emetic and enteropathogenic *B. cereus* strain. The highest *cesB* expression in our tested samples was measured after nine hours of cultivation, significantly dropping at the 15 h time point. This aligns with previous work indicating that cereulide production in the transition phase is repressed by the transition state regulator AbrB at later growth phases [61,71]. As expected, no *cesB* transcripts were observed in the enteropathogenic *B. cereus* since it does not harbor the *ces* gene locus [70]. In contrast, the chromosomally encoded virulence factors *nhe* and *sph*, which contribute to enteropathogenicity, and *plc* were transcribed in both the enteropathogenic and emetic strains, although at different levels. It has been reported previously that the actual enteropathogenic potential of a particular *B. cereus* strain is linked to the expression level of Nhe and SMase [16,72].

There is a close biological linkage between enterotoxins and phospholipases in *B. cereus*. First, they act synergistically, enhancing the overall pathogenic potency of a given strain [28,29]. Second, the phospholipases SMase and PC-PLC can be shuttled together with enterotoxin components by extracellular vesicles to host cells in a protected manner [73]. Third, the gene expression of Nhe, SMase and PC-PLC is driven by the same pleiotropic transcriptional regulator PlcR [35,36].

PlcR, which is activated by the quorum-sensing molecule PapR [74,75], is the master quorum-sensing regulator in enteropathogenic *B. cereus*. In its processed form, PapR can specifically interact with PlcR and facilitate the binding of PlcR to its DNA targets. In contrast, the disruption of *papR* abolishes the expression of genes belonging to the PlcR regulon [39,76,77]. Using our newly established set of RT-qPCR reference genes, we could verify similar gene expression patterns of the aforementioned virulence factors in both environments, LB and MOD. Since these virulence factors belong to the PlcR regulon, the observed similarities in their expression pattern were to be expected and might, at least partially, be related to their transcriptional control by the quorum-sensing regulator PlcR.

The PlcR-regulated genes *sph*, *nheB* and *plc* were shown to be downregulated over time, with the highest transcript levels in the early growth phase. These data corroborate previous transcription studies showing that *plcR* and, subsequently, *nheB* mRNA levels decrease over time [16,71]. Although the transcriptional patterns in LB and MOD were comparable for *sph* and *plc*, the kinetics of *nheB* expression differed between both media, highlighting the impact of extrinsic factors on gene expression. It has been shown previously that distinctive growth environments have a significant impact on the kinetics and the amount of enterotoxins produced by a particular strain [78]. In our current study, environmental challenges were mimicked by cultivating *B. cereus* in the full medium LB or the minimal medium MOD. The inherently different compositional media profiles lead to different nutrient availabilities and osmolarities, which may impact the expression of virulence factors. Changes in the salt concentration of the cultivation medium have previously been revealed by Dommel et al., 2011, to affect cereulide production [21]. As reported by Jessberger et al., 2017, the Nhe toxin was detectable in bacterial cultures grown under host-mimicking conditions as early as two hours. These findings are in line with the results from our newly established RT-qPCR, demonstrating that the maximal expression level of *nheB* is reached earlier in MOD than in LB.

The biological differences between the toxigenic reference strains associated with emetic and enteropathogenic syndromes were particularly evident in the regulation of *nheB*, which varied significantly between the strains F4810/72 and NVH0075-95. We have previously shown that the branched-chain amino acid-sensing regulator CodY acts as a nutrient-responsive transcription regulator that orchestrates cereulide and enterotoxin synthesis in *B. cereus* F4810/72 [71]. Furthermore, a detailed analysis of the promoter region of *B. cereus* enterotoxin genes by Böhm et al., 2016, suggests that the low conservation of CodY binding sites in *B. cereus* group strains may contribute to the strain-specific modulation of toxin synthesis [79].

In summary, the results from our proof-of-principle study highlight the complexity of multilayer networks involved in the transcriptional and posttranscriptional regulation of enterotoxins and virulence factors in *B. cereus*, which are far from understood.

Thus, our newly established set of reference genes, including *rho*, *rpoD* and *recA*, is expected to facilitate data normalization in future RT-qPCR-based studies to exploit the intricate multifaceted cellular processes that orchestrate the panoply of toxins and other virulence factors in pathogenic *B. cereus*. Understanding the molecular mechanisms behind toxin regulation could pave the way for innovative strategies to harness the beneficial properties of *B. cereus*, while mitigating its pathogenic potential. Insights into the regulatory pathways coining toxin production could also potentially be leveraged toward new pharmaceutical strategies to treat bacterial infections.

## 4. Materials and Methods

### 4.1. Bacterial Cultivation and RNA Isolation

Two toxigenic *B. cereus* strains, the emetic reference strain F4810/72 and enteropathogenic reference strain NVH0075-95, were used as model organisms. Both strains included in this study have been isolated in the context of foodborne outbreaks. *B. cereus* F4810/72 (also known as AH187) was implicated in a foodborne outbreak in 1972 in the UK. It was isolated from the vomit of a patient after the consumption of contaminated rice by the Public Health Laboratory Service (PHLS) in London, UK. It has been extensively characterized by Ehling-Schulz et al. [70]. It is the commonly known reference strain for the emetic type of *B. cereus* because it was used to decipher the molecular basis of the non-ribosomal synthesis of the emetic toxin cereulide [17,20].

*B. cereus* NVH0075-95 was isolated from stew with vegetables, which was implicated in a large foodborne outbreak in Norway in 1995 [27]. NVH0075-95 is a commonly used reference strain for the enteropathogenic type of *B. cereus* since the non-hemolytic tripartite enterotoxin Nhe, a key virulence factor in enteropathogenic *B. cereus*, was first described and characterized in this strain [27].

Bacteria were pre-cultured in 3 mL LB and incubated for 8 h at 30 °C while shaking at 120 rpm. These pre-cultures were used to inoculate the 100 mL main cultures with a start OD_600_ of 0.05. The main cultures were incubated at 30 °C while shaking at 120 rpm for three, nine and fifteen h. LB-Miller broth (LB) and modified optimal defined medium (MOD) liquid media were used for the main cultures [45,46]. LB contains 10 g tryptone, 5 g yeast extract and 10 g NaCl per liter. MOD medium [45] consists of a basic medium [45.41 mM (NH_4_)_2_SO_4_, 5.74 mM K_2_HPO_4_, 0.16 mM MgSO_4_] supplemented with trace elements (3.39 mM FeCl_2_ × 6 H_2_O, 0.25 mM MnCl_2_ × 4 H_2_O, 0.12 mM Na_2_MoO_4_ × 2 H_2_O, 2.47 mM CaCl_2_, 0.62 mM ZnCl_2_, 0.12 mM CoCl_2_ × 6 H_2_O, 0.25 mM CuSO_4_, 0.12 mM NaSeO_4_), 20 mM D-glucose and a mixture of 15 amino acids (13.60 mM L-glutamic acid, 5.20 mM L-glycine, 7.77 mM L-valine, 7.64 mM L-threonine, 2.68 mM L-methionine, 2.32 mM L-histidine, 2.64 mM L-arginine, 6.83 mM L-aspartic acid, 0.33 mM L-cysteine, 5.37 mM L-isoleucine, 10.44 mM L-leucine, 1.70 mM L-phenylalanine, 8.07 mM L-lysine, 6.28 mM L-serine, 0.23 mM L-tyrosine). The composition of both media in a table format is shown in Supplementary Table S2.

Transcription of toxin genes and candidate reference genes in both *B. cereus* strains was analyzed by RT-qPCR as described previously [80]. For RNA extraction, the main cultures were centrifuged at 5,000 × *g* for 5 min to obtain the cell pellets. The supernatant was discarded, and the cell pellets were snap-frozen in liquid nitrogen and stored at −80 °C until RNA extraction.

RNA was extracted from the frozen pellets using TRIzol reagent (Thermo Fisher, Waltham, MA, USA) and homogenized using a FastPrep^®^-24 Ribolyser (MP Biomedicals, Santa Ana, CA, USA) at a speed of 6.5 for 2 cycles of 45 s each, with 30 s on ice between cycles. ZnSn beads (0.1 mm) in 2 mL screw-top tubes were used to homogenize the samples. Chloroform was added to induce phase separation, and nucleic acids were precipitated with 75% ethanol. The RNA concentration was measured using a Nanodrop™ 2000 spectrophotometer (Thermo Fisher, Waltham, MA, USA). Samples were then diluted to achieve a final volume of 2 µg for cDNA synthesis using the iScript™ gDNA Clear Synthesis Kit (Bio-Rad, Hercules, CA, USA).

### 4.2. Primer Design and Efficiency Testing

All primers except for *cesB* were designed specifically for this study. Primers targeting *cesB* (Table 1) were designed based on the plasmid sequence (GenBank accession number DQ360825, see Table 1 for localization) [21]. Primers for this study were designed using the Primer3 algorithm [81,82,83] with the following parameters: primer length of 21 ± 3 bp, GC content of 40–60% and a melting temperature (T_m_) of 60 ± 0.5 °C. Primers were designed using the genomes of F4810/72 [84] (GenBank accession number: GCA_000021225.1) and NVH0075-95 [27] (GenBank accession number: GCF_027945115.1) as reference genomes, ensuring complete sequence identity with both strains (for localization in F4810/72, see Table 1; for detailed gene accession numbers, see Table S1). Primer integrity and potentially emerging secondary structures were further assessed using NetPrimer (Premier Biosoft, San Francisco, CA, USA) [85,86,87]. RT-qPCR efficiencies of various primer sets were calculated by using seven serial dilutions of pooled cDNA as previously described [21]. By plotting Cq values and the corresponding negative logarithm of the dilution factor, a linear regression model was calculated, and both Pearson’s correlation coefficient (R^2^) and the respective slope were determined. RT-qPCR efficiencies E were calculated according to the following equation.

(1) $$E\left(\%\right)=\left(10^{-\;\frac{1}{\mathrm{Slope}}}-1\right)\times \;100$$

### 4.3. RNA Quantification by RT-qPCR

For the gene expression analysis of cellular RNA, qPCR reactions were performed using the SsoFast EvaGreen Supermix (Bio-Rad, Hercules, CA, USA) on a Rotor-Gene Q thermal cycler (model 5-Plex HRM, Qiagen, Hilden, Germany) in a total volume of 10 μL, with 1 ng cDNA and a respective primer concentration of 500 nM. The thermal cycling protocol included an initial polymerase activation step at 95 °C for 30 s, followed by 40 cycles of denaturation at 95 °C for 5 s, and annealing at 57 °C for *cesB* and 60 °C for all other genes for 5 s. Controls without a template were included to verify the absence of secondary structures of primers. Rotor-Gene Q 2.3.1.49 software (Qiagen, Hilden, Germany) was used to obtain Cq values, setting the threshold for quantification in the exponential phase.

### 4.4. Data Analysis

A thorough literature search identified potentially suitable reference genes for data normalization [57,58,59,88]. After obtaining the RT-qPCR data of the candidate reference genes, RefFinder [56] was employed to obtain a comprehensive ranking of four commonly used algorithms: BestKeeper [47], NormFinder [53], geNorm [54] and the comparative ΔCq method [55]. This ranking was used to identify the most stable combination of reference genes. For further normalization of gene expression data, the geometric mean of the most robust combination—*rho*, *rpoD* and *recA*—was used.

The ∆∆Cq method [89] was used to calculate target gene expression data. The arithmetic mean of the Cq values from two technical replicates per sample was calculated, and the geometric mean of the resulting Cq values of the reference gene combination (*rho*, *rpoD* and *recA*) was subtracted to calculate ∆Cq values. The ∆Cq values were then normalized to the respective reference conditions to obtain ∆∆Cq values. Statistical significance was determined between ∆∆Cq values retrieved from samples from the same culture media by two-way ANOVA (Figure 4) or one-way ANOVA (Figure 5) with Tukey‘s correction for multiple testing. Statistical significance regarding strain-dependent differences in Cq values of reference gene expression (Figure S1A) was determined by multiple *t*-tests with Holm–Sidak correction for multiple testing. Data visualization and statistical analysis were performed using GraphPad Prism 10.4.0.

## Figures and Tables

**Table 1:** Oligonucleotide primers designed for RT-qPCR.

Gene Name	Product Name	Primer Sequences (5′–3′)	Nucleotide Position	Product Size (bp)	E (%)	R^2^
*cesB*	cereulide synthetase B	TTAGATGGTATTCTTCACTTGGC	20,181–20,203	308	114.24	0.9961
		TTGATACAAATCGCATTCTTATAACC	20,463–20,488			
*nheB*	non-hemolytic enterotoxin NHE subunit B	CTTTAGTTGCTGCGGTAGATGC	1,859,686–1,859,707	168	91.02	0.9961
		CATCACCCTTGAAGTTTTGCGT	1,859,832–1,859,853			
*sph*	sphingomyelinase C	ATTGGGGACAAAGTCAGCGT	762,343–762,362	116	95.29	0.9927
		TCCTAAAAGGCGATCTGAAGCA	762,437–762,458			
*plc*	phospholipase C	GAACGGTATTTATGCTGCTGACT	761,515–761,537	125	102.78	0.9933
		CAGTTTCTTTTGCCTGCTTTGC	761,618–761,639			
*gatB_* *Yqey*	gatB/Yqey domain- containing protein	GTCATTCGTATGGTTAAGGCTGC	4,115,261–4,115,283	223	103.84	0.9969
		GCTCTTCTTCCGTTAATTGCTCC	4,115,061–4,115,083			
*gyrA*	DNA Gyrase subunit A	AAGAGGTTACCAGCTTCCACGT	8148–8169	72	93.54	0.997
		ATTTGTCCCCATTCCTTGCAC	8199–8219			
*proC*	Pyrroline-5-carboxylate reductase	GATCGCTGCTGGTAAAAGTATTGA	2,802,477–2,802,500	136	113.93	0.9467
		TGTCACCATTTCATTCGGGC	2,802,593–2,802,612			
*recA*	recombinase A	CACCACCATTCCGTGTTGC	3,578,587–3,578,605	151	88.98	0.988
		CGACCTTGTCCTAAGCGTTCT	3,578,455–3,578,475			
*rho*	transcription termination factor Rho	ACGACCTCCGAAAGAAAATGAAC	5,093,968–5,093,990	125	94.6	0.9917
		TGGCGATCTGGGTATAATGGTG	5,093,862–5,093,883			
*rpoD*	RNA polymerase sigma factor RpoD	CACAGGAGCCAGTTTCTCTTGA	4,101,300–4,101,321	91	102.88	0.9921
		GGCGATGTTGCTTCTTGGTC	4,101,231–4,101,250			
*rpsU*	30S ribosomal protein S21	AGATCGGTTTCTAAAACTGGTACAC	4,115,460–4,115,484	111	99.93	0.9958
		GAATTTACGCTTTCTTGCCGC	4,115,374–4,115,394			
*rrn*	16S rRNA	TTTCCGCCCTTTAGTGCTGA		250	120.31	0.9975
		CCCAACATCTCACGACACGA				
*wecB*	UDP-N-acetylglucosamine 2-epimerase	GCAGGACTTCGTACATGGGAT	4,946,363–4,946,383	79	101.39	0.9962
		TCGTTGCTGATTTTGCTGTAGG	4,946,272–4,946,293			

## Data Availability

The original contributions presented in this study are included in the article/Supplementary Materials. Further inquiries can be directed to the corresponding author.

## Supplementary Materials

The following supporting information can be downloaded at: https://www.mdpi.com/article/10.3390/toxins17020058/s1, Figure S1: Comparison of Cq values of candidate reference genes of emetic and enteropathogenic *B. cereus* strains; Table S1: Accession numbers of candidate reference genes for accurate gene expression profiling in toxigenic *Bacillus cereus*; Table S2: Media composition of MOD medium and LB-Miller broth.
