# Supplementary material for: Establishing Reference Genes for Accurate Gene Expression Profiling in Toxigenic Bacillus cereus
Source: Toxins (Basel). 2025 Jan 27;17(2):58. doi: 10.3390/toxins17020058 (PMC11860165; doi:10.3390/toxins17020058)
Supplement: Supplementary file 1 [file toxins-17-00058-s001.zip › toxins-3394902-supplementary.pdf]

toxins

MDPI

Supplement

Establishing Reference Genes for Accurate Gene Expression Profiling in Toxigenic *Bacillus cereus*

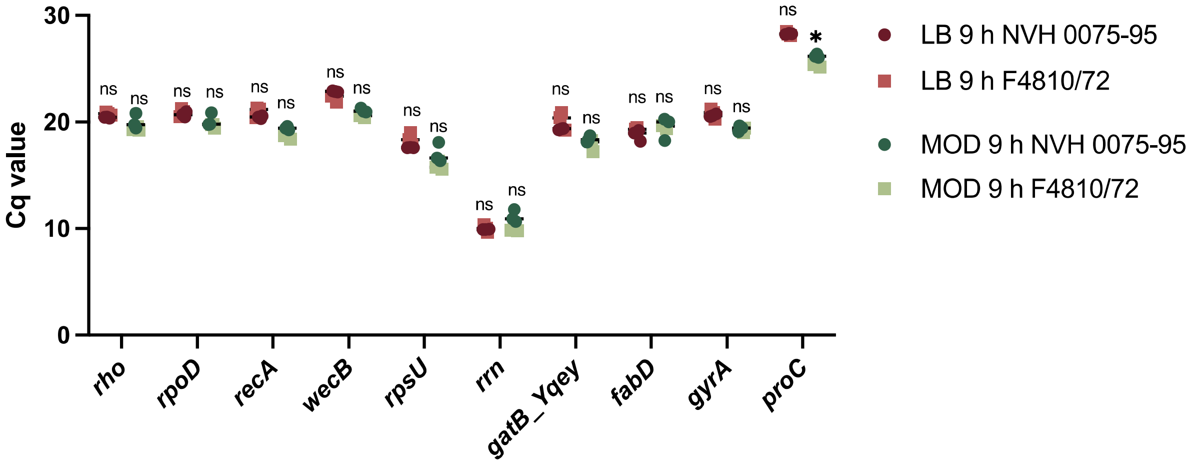

Fig.S1: Comparison of Cq values of candidate reference genes of emetic and enteropathogenic *B. cereus* strains

Cq values derived from *B. cereus* F4810/72 and *B. cereus* NVH0075-95 grown in LB or MOD for 9 hours are shown for the reference genes evaluated in this study. Statistical significance was calculated using multiple t-tests with Holm-Sidak correction for multiple testing. Statistical significance levels are indicated as follows: ns > 0.05, *p < 0.05.

Supplementary Table 1: Accession numbers of candidate reference genes for accurate gene expression profiling in toxigenic *Bacillus cereus*

Gene Name	Product Name	Gene ID F4810/72	Gene ID NVH0075-95
fabD	Malonyl CoA-acyl carrier protein transacylase	BCAH187_A3900	PGS39_RS19190
gatB_Yqey	gatB/Yqey domain-containing protein	BCAH187_A4441	PGS39_RS22145
gyrA	DNA Gyrase subunit A	BCAH187_A0006	PGS39_RS00030
proC	Pyrroline-5-carboxylate reductase	BCAH187_A3029	PGS39_01140
recA	recombinase A	BCAH187_A3826	PGS39_RS18830
rho	transcription termination factor Rho	BCAH187_A5510	PGS39_RS27250
rpoD	RNA polymerase sigma factor RpoD	BCAH187_A4426	PGS39_RS22075
rpsU	30S ribosomal protein S21	BCAH187_A4442	PGS39_RS22150
rrn	16S rRNA		
wecB	UDP-N-acetylglucosamine 2-epimerase	BCAH187_A5365	PGS39_RS26545

Supplementary Table 2: Media composition of MOD medium and LB-Miller broth, according to Rosenfeld *et al.*, 2005 [45] and Miller, J.H. 1972 [46].

MOD media composition

pH	7.2
Osmolarity	0.240 mOsmol

Carbon source	*[mmol/l]*
D-(+)-glucose (Carl Roth, Karlsruhe, Germany)	30

Amino acids	*[g/l]*
L-glutamic acid (Carl Roth, Karlsruhe, Germany)	2
L-glycine (Carl Roth, Karlsruhe, Germany)	0,39
L-valine (Carl Roth, Karlsruhe, Germany)	0,91
L-threonine (Carl Roth, Karlsruhe, Germany)	0,91
L-methionine (Carl Roth, Karlsruhe, Germany)	0,4
L-histidine (Carl Roth, Karlsruhe, Germany)	0,36
L-arginine (Carl Roth, Karlsruhe, Germany)	0,46
L-aspartic acid (AppliChem, Darmstadt, Germany)	0,91
L-cysteine (Carl Roth, Karlsruhe, Germany)	0,04
L-isoleucine (Carl Roth, Karlsruhe, Germany)	0,7
L-leucine (Carl Roth, Karlsruhe, Germany)	1,37
L-phenylalanine (Carl Roth, Karlsruhe, Germany)	0,28
L-lysine (Sigma-Aldrich, USA)	1,18
L-serine (Carl Roth, Karlsruhe, Germany)	0,66
L-tyrosine (Carl Roth, Karlsruhe, Germany)	0,042

Basic medium	*[g/l]*
$(NH_4)_2SO_4$ (Carl Roth, Karlsruhe, Germany)	6
$MgSO_4$ x 7 H_2O (Carl Roth, Karlsruhe, Germany)	0,04
K_2HPO_4 (Carl Roth, Karlsruhe, Germany)	1

Trace elements	*[µg/l]*
$FeCl_2$ x 6 H_2O (Sigma-Aldrich, Saint Louis, MO, USA)	675
$MnCl_2$ x 4 H_2O (Carl Roth, Karlsruhe, Germany)	50
Na_2MoO_4 x 2 H_2O (Carl Roth, Karlsruhe, Germany)	30
$CaCl_2$ (Carl Roth, Karlsruhe, Germany)	275
$ZnCl_2$ (Carl Roth, Karlsruhe, Germany)	85
$CoCl_2$ x 6 H_2O (Carl Roth, Karlsruhe, Germany)	30
$CuSO_4$ (Carl Roth, Karlsruhe, Germany)	40
Na_2SeO_4 (Carl Roth, Karlsruhe, Germany)	24

LB-Miller broth composition

pH	7.4
Osmolarity	0.429 mOsmol

Component	*[g/l]*
NaCl (Carl Roth, Karlsruhe, Germany)	10
Tryptone (Oxoid, Basingstoke, UK)	10
Yeast extract (Oxoid, Basingstoke, UK)	5
